# Porvac^®^ Subunit Vaccine E2-CD154 Induces Remarkable Rapid Protection against Classical Swine Fever Virus

**DOI:** 10.3390/vaccines9020167

**Published:** 2021-02-17

**Authors:** Yusmel Sordo-Puga, Marisela Suárez-Pedroso, Paula Naranjo-Valdéz, Danny Pérez-Pérez, Elaine Santana-Rodríguez, Talia Sardinas-Gonzalez, Mary Karla Mendez-Orta, Carlos A. Duarte-Cano, Mario Pablo Estrada-Garcia, María Pilar Rodríguez-Moltó

**Affiliations:** 1 Animal Biotechnology Department, Center for Genetic Engineering and Biotechnology, P.O. Box 6162, Havana 10600, Cuba; yusmel.sordo@cigb.edu.cu (Y.S.-P.); marisela.suarez@cigb.edu.cu (M.S.-P.); danny.perez@cigb.edu.cu (D.P.-P.); elaine.santana@cigb.edu.cu (E.S.-R.); talia.sardina@cigb.edu.cu (T.S.-G.); mary.mendez@cigb.edu.cu (M.K.M.O.); carlos.duarte@cigb.edu.cu (C.A.D.); mario.pablo@cigb.edu.cu (M.P.E.); 2 Central Laboratory Unit for Animal Health (ULCSA), Havana 11400, Cuba; labconestatal@hab.minag.cu

**Keywords:** classical swine fever virus, Porvac^®^ subunit vaccine E2-CD154, early protection, T cell IFNγ responses

## Abstract

Live attenuated C-strain classical swine fever vaccines provide early onset protection. These vaccines confer effective protection against the disease at 5–7 days post-vaccination. It was previously reported that intramuscular administration of the Porvac^®^ vaccine protects against highly virulent classical swine fever virus (CSFV) “Margarita” strain as early as seven days post-vaccination. In order to identify how rapidly protection against CSFV is conferred after a single dose of the Porvac^®^ subunit vaccine E2-CD154, 15 swine, vaccinated with a single dose of Porvac^®^, were challenged intranasally at five, three, and one day post-vaccination with 2 × 10^3^ LD_50_ of the highly pathogenic Cuban “Margarita” strain of the classical swine fever virus. Another five animals were the negative control of the experiment. The results provided clinical and virological data confirming protection at five days post-vaccination. Classical swine fever (CSF)-specific IFNγ T cell responses were detected in vaccinated animals but not detected in unvaccinated control animals. These results provided the first data that a subunit protein vaccine demonstrates clinical and viral protection at five days post-vaccination, as modified live vaccines.

## 1. Introduction

Classical swine fever (CSF) is considered to be a devastating disease for the pig industry throughout the world concerning both economic and sanitary issues [1]. The causative agent of CSF, which is a highly contagious disease of swine, is a member of the genus *Pestivirus* within the family *Flaviviridae*. Classical swine fever virus (CSFV) has a positive-sense, single-stranded RNA genome that is contained by an enveloped viral capsid [2]. Huge economic losses are caused by CSF outbreaks, due to impaired production and the disruption of the internal and international trade of pigs and pig-products [1,3]. The slaughter of suspected and infected herds, or vaccination with attenuated CSFV strains, are the more frequent procedures for CSF control. Although the currently available CSFV live attenuated viruses (LAVs) confer an effective, rapid, and solid immune protection, countries that are free of CSFV do not apply vaccination to their national herds due to the inherent difficulty to differentiate infected animals within a vaccinated population (i.e., Differentiating Infected from Vaccinated Animals (DIVA) capability). Live attenuated C-strain CSFV vaccines provide complete protection in at least seven days, but the lack of DIVA is problematic for eradication programs in CSF enzootic areas [4]. Some live attenuated vaccines also carry genuine concerns over their safety, due to the potential of reversion to virulence and recombination with pathogens in the field [5,6]. 

Porvac^®^ subunit E2-CD154 is a novel, DIVA vaccine that is a chimeric protein formed by the fusion of the extracellular region of E2 glycoprotein of CSFV “Margarita” strain and the extracellular segment of swine CD154 molecule The immunogenicity and thermal stability demonstrated by this subunit vaccine make it a potent tool for use in the control and eradication programs of the CSFV, especially in developing countries, where the field conditions require a vaccine that can overcome the known inconveniences that are typically associated with the traditional use of LAVs in diverse farm settings. 

Vaccination with C-strain vaccines induces both neutralising antibody and T cell responses. Studies have shown that C-strain conferred protection may occur prior to the onset of antibody induction and, so, virus-specific T cell responses have been implicated in mediating the early protection under such circumstances [7]. There is also a close temporal correlation between T cell IFNγ responses and rapid protection that is induced by a C-strain vaccine five, three, or one day prior to challenge infection [8,9]. 

Marker sub-unit vaccines that are based upon the major viral envelope glycoprotein E2 have been licensed, but the requirement for two vaccinations four weeks apart to ensure protection has limited their usage under outbreak conditions [10]. For newly developed vaccines that could be considered for emergency vaccination, the fast onset of immunity is of paramount importance, as it will stop virus spread and lead to fast control and eradication of the disease [11]. 

Previously, a complete clinical and viral protection seven days after receiving the first dose of E2CD154 was reported [12,13]. The aim of this study was to identify how rapid protection against CSFV is conferred after a single dose of the Porvac^®^ subunit vaccine E2-CD154.

## 2. Materials and Methods 

### 2.1. Porvac^®^ Subunit Vaccine E2-CD154

Porvac^®^ is a chimeric protein that is formed by the fusion of the extracellular region of E2 glycoprotein of CSFV “Margarita” strain (aa 2-364, nt 4-1092 from GenBank Accession Number AJ704817), and the extracellular segment of swine CD154 molecule (210 aa, GenBank Accession Number: AB040443). A lentivirus-based gene delivery system was used to generate a stable recombinant HEK 293 cell line (ATCC CRL1573) for the expression of E2-CSFV antigen fused to porcine CD154 molecule, as previously described [12]. E2CD154 protein was formulated in MontanideTM ISA50 V2 (SEPPIC, La Garenne-Colombes, France) while using a 60/40 proportion of aqueous/oil phase. The “water in oil” emulsion was produced with a SD-41 homogenizer (IKA, Königswinter, Germany) under Good Manufacturing Practice (GMP) conditions. The concentration of E2CD154 in the final emulsion was 25 μg/mL. 

### 2.2. Experimental Animals 

The trials were carried out under appropriate high containment conditions following the animal welfare regulations and standards according to EU Directive 2010/63/EU (EU Directive 2010/63, Official J. of EU) and Good Clinical Practices (VICH GL9 (GCP) June 2000). Nine-week-old Crossbred Duroc/Yorkshire swine (25–30 kg) belonging to a non-vaccinated and CSF-free herd were used. (CENPALAB, National Centre for Animal Production, Cuba). The animals were fed with 2 Kg/per animal/per day and water ad libitum. Vaccinated animals were carefully evaluated daily for clinical signs, inappetence, prostration, inflammatory reactions at the inoculation site, appreciable changes in respiratory rate, or other alterations that may be related, or not, to vaccination.

A total of 20 swine were used. The pigs were tagged in the paddocks with visible identification (notches) and they were allocated at random into four experimental groups each of five animals

### 2.3. Immunization Schedule and Experimental Design

Control animals (Group 1) were immunized with mock formulation (Montanide ISA50V2, SEPPIC, France) five days before viral challenge. The animals were immunized once by intramuscular injection with 2ml of formulation (25ug/mL) (50 µg of purified E2CD154 antigen). Piglets were challenged five (Group 2), three (Group 3), or one (Group 4) days post-vaccination (dpv). All of the groups were challenged Intranasally (IN) with 2 × 10^3^ LD_50_ of CSFV high virulent “Margarita” strain, genotype 1.4 [14]. Clinical signs were scored daily for 28 days after challenge according to Mittelholzer et al., [15] with modifications. The modifications consist in the elimination of three parameters (body tension, body shape, and breathing) and the addition of the rectal temperature. The samples of heparinized blood and serum were taken at the moment of challenge, and at 3, 7, 14, 18, 21, and 28 days post-challenge. Blood samples were taken by ophthalmic venous sinus punctures using sterile tubes, with and without anti-coagulant (VACUATTE^®^ Greiner bio-one, Frickenhausen, Germany). The blood samples were placed at room temperature for 2 h and then kept overnight at 2–8 °C to allow for serum extraction. 

### 2.4. Neutralizing Antibodies Detection

The serum samples were screened for the ability to neutralize the cell culture adapted “Margarita” CSFV strain (National Center for Animal and Plant Health, Mayabeque Cuba) while using neutralization peroxidase-linked assay (NPLA) [16], as described in the Manual of World Organization for Animal Health [17]. After inactivating, the serum was diluted in two-fold steps with a cell growth medium and an equal volume of a neutralizing test viral solution containing approximately 100 TCID/50 in 50 μL was added to each diluted serum and then incubated at 37 °C for 60 min. A quantity of 50 µL of PK15 cells suspended at 8 × 10^4^ cell/mL were added and incubated at 37 °C with 5% CO2 for 72 h and then observed. The virus dilution, covering a range of four logarithms, was added to the neutralization plates and then subjected to reverse titration. Back titration, which acts as an internal quality control, confirmed that the virus was used in a concentration between 30 and 300 TCID50/50 μL. Virus neutralization was detected by incubation with the anti-E2 Mab CBSSE2.3 (CIGB-SS, Cuba) conjugated to horseradish peroxidase and then incubated with 3-amino-9-ethyl carbazole (AEC) and hydrogen peroxide. The presence of viral replication was determined by visual inspection with an optical microscope. Titers were expressed as the reciprocal of the highest dilution of serum that neutralized 100 TCID50 of “Margarita” strain in 50% of culture replicates. The results were expressed as the geometric mean (GM) of the NAb titers plus the confidence intervals.

### 2.5. Viral Isolation 

For viral isolation, heparinized blood samples were taken at day 3, 7, 14, 21, and 28 days post-challenge (dpc). Tonsils and spleens were collected at the end of the experiment at 28 dpc. Tissues (approximately 1 cm^2^) were macerated in 1 mL of DMEM (Sigma, St. Louis, MO, USA) supplemented with 5% fetal calf serum, penicillin (100 IU), and streptomycin (100 μg). The homogenates were resuspended in 4 mL of DMEM and allowed to settle for 1h at room temperature. Afterward, the samples were centrifuged at 1200 rpm for 15 min. and the supernatant transferred and preserved in cryovials (Sigma—Aldrich, St. Louis, MO, USA) at −80 °C. Viral isolation was performed in PK15 cells through two serial passages in 48 wells microplates and the third passage onto 96 wells plates, six replica wells for each sample. Virus was detected with the anti-E2 Mab CBSSE2.3 (CIGB-SS, Cuba) that wsa conjugated to horseradish peroxidase, followed by incubation with 3-amino-9-ethyl carbazole (AEC) and hydrogen peroxide according to the protocol that was described in the Manual of World Organization for Animal Health [17].

### 2.6. Elispot Assay for Detection of CSFV-Specific IFN-γ Producing Cells

At the moment of challenge, peripheral blood mononuclear cells (PBMC) were isolated by density-gradient centrifugation using Histopaque 1077 (Sigma, St. Louis, MO, USA ). The total number of live PBMC recovered was estimated by trypan-blue staining and counting in Neubauer chamber. An Elispot assay was performed using 96 well plates (Millipore, Merck KGaA, Darmstadt, Germany ) and porcine IFN-γ ELISpot BASIC kit (MABTECH, Nacka Strand, Sweden), according to the manufacturer´s instructions. Briefly, the plates were coated overnight with capture antibody and then 5 × 10^5^ PBMC/well were plated in triplicate in the presence of either 0.05 MOI of CSFV Margarita strain or 5 μg/mL of E2CD154. As controls, cells were incubated in triplicate in the absence of virus (negative control) or with phytohaemagglutinin (PHA) (10 μg/mL) as positive control. The frequencies of antigen-specific IFN-γ producing cells were calculated by subtraction of spots in the non-stimulated wells from the spots in CSFV stimulated wells, and they were expressed as number of responder cells in 2 × 10^5^ PBMC.

### 2.7. Statistical Analysis 

The normal distribution of the data was assessed by Kolmogorov–Smirnov and D’Agostino–Pearson tests. The Kruskal–Wallis test was used to compare antibody titers and IFN-γ producing cells among groups of animals and Dunn’s multiple comparisons test to look for individual differences among groups. The statistical package GraphPad Prism 6 was used for all of the analysis (Prism 6 for Windows, Version 6.01, GraphPad Software, Inc., La Jolla, CA, USA). Statistical significance was considered when *p* < 0.05.

## 3. Results

### 3.1. Vaccination Five Days Prior to Challenge Protected Animals Against Clinical Disease and Prevented Infection

**Figure 1:**
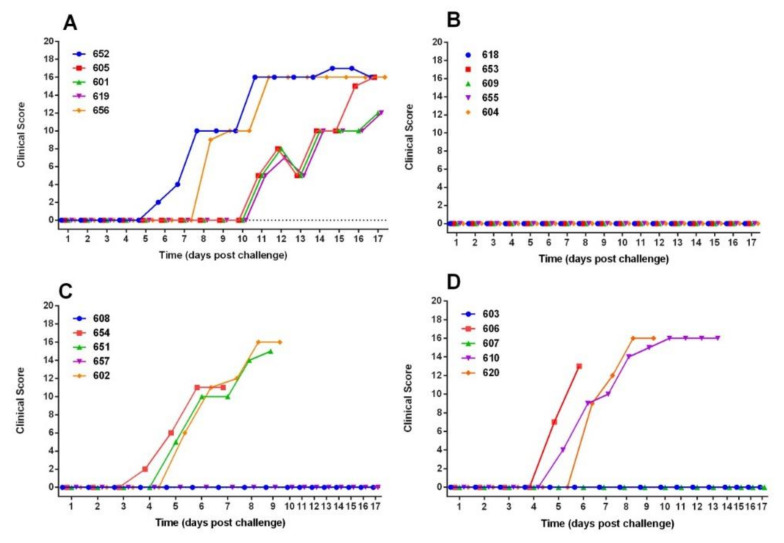
Clinical Score after intranasal challenge with “Margarita” strain of CSFV. (**A**) Group 1: Control animals, (**B**) Group 2: Animals immunized once five days before challenge, (**C**) Group 3: Animals immunized once three days before challenge, and (**D**): Group 4: Animals immunized once one day before challenge.

**Figure 2:**
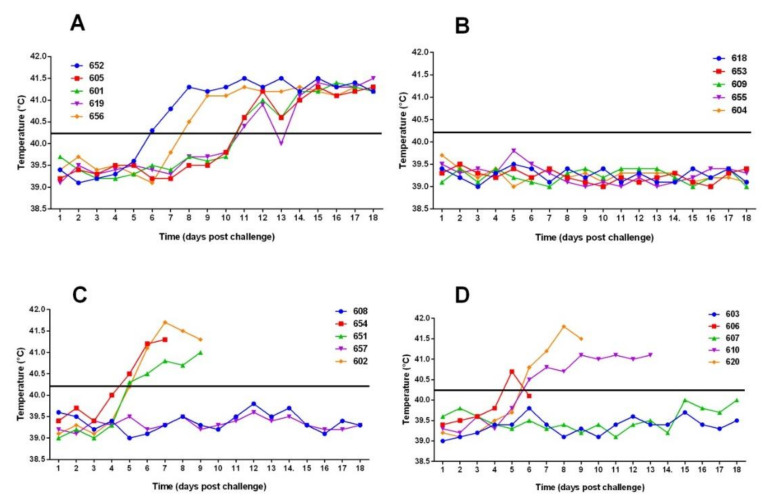
Rectal temperatures of animals post-challenge with “Margarita” strain of CSFV. (**A**) Group 1: control animals (**B**) Group 2: animals immunized once five days before challenge. (**C**) Group 3: animals immunized once three days before challenge. (**D**) Group 4: animals immunized once one day before challenge. The line represents the value of 40.2 °C, temperatures above which were considered as a sign of fever.

Two animals (652 and 656) in the control group showed clinical signs from days five and six post-confrontation, coinciding with an increase in temperature greater than 40.4 °C (Figure 1 and Figure 2). The clinical signs were observed later in the three remaining control animals (605, 601 and 619), starting from day 11 post-challenge. In this group, the animals were euthanized at 18 days post-challenge for ethical reasons. At the moment of euthanasia, temperatures reached average values of 41.3 ± 0.14. Anorexia, ataxia, diarrhea, conjunctivitis, respiratory disorders, severe prostration, and nervous symptoms were observed in the animals. Post-mortem examination revealed specific lesions of CSFV infection (suppurative tonsillitis, marginal splenic infarction, generalized bleeding, and others) and the virus was isolated from blood and tissues (Table 1). 

Animals that were vaccinated five days prior to challenge (Group 2) had no clinical signs of the disease and a normal temperature response was observed in all animals (Figure 1 and Figure 2). The virus was detected in blood at 3dpc only in one animal (No. 655) in this group. However, no virus was isolated from the tonsils, spleen, ileum, and rectal exudate at post-mortem (28 dpc) from this animal. No virus was detected in the tissues or rectal swabs at euthanasia of the rest of the animals of this group, except for animal 618, which was virus positive in ileum but not in internal organs (Table 1). In addition, no macroscopic pathological lesions that were compatible with CSF were observed at 28 dpc in any of the animals vaccinated five days prior to challenge.

Three of the five animals in each of the groups vaccinated three or one day before challenge developed clinical signs at day 4–5 post-challenge. In group 3 (challenged 3 pdv), the animals 654, 651, and 602 had temperature values over 40.2°C starting on the fifth day post-challenge. In group 4 (challenged 1 dpv), the animals 606, 610, and 620 show temperatures values over 40.0 °C, starting at the fifth day post-challenge. These affected animals were euthanized between seven at 13 days post-challenge for ethical reasons, with 16 point in the clinical score. In both of these groups, two of five animals (60%), remained without clinical signs for the duration of the study (Figure 1 and Figure 2). The animals affected in both the dpv 1 and dpv 3 groups had virus in blood and organs, especially tonsil and spleen (Table 1). In contrast, no virus was detected in the blood or tissues of animals that remained healthy. 

**Figure 3:**
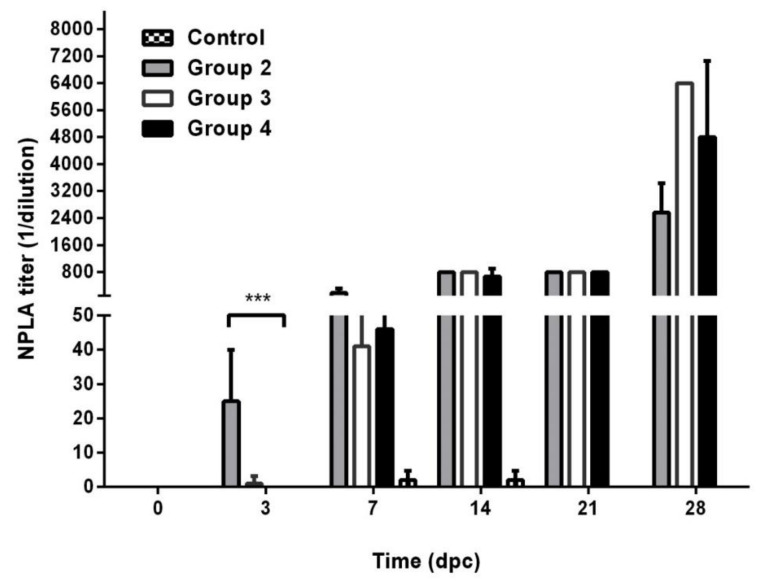
Neutralizing antibodies titres. Bars represent the geometric mean of the antibody titres plus the 95% confidence interval. Group 1: control animals. Group 2: animals immunized once five days before challenge. Group 3: animals immunized once three days before challenge. Group 4: animals immunized once one day before challenge. Dpc: days post-challenge. *** *p* < 0.001

At the time of challenge, neutralizing antibodies were not detected in either the vaccinated or control animals (Figure 3), demonstrating that the innate immune response has a role in protection from the challenge. Three days post-challenge, the animals in the 5 dpv group, which were protected from challenge, had detectable neutralizing antibodies (mean titers of 1:25 ± 15). In the animals vaccinated three or one days before challenge neutralizing antibodies titres were statistically lower (mean titres ≤1:5, Kruskal–Wallis test, *p* = 0.0009; Dunn test *p* < 0.05) at the same time post-challenge (six and four days post-vaccination, respectively).

### 3.2. IFN Gamma Determination 

**Figure 4:**
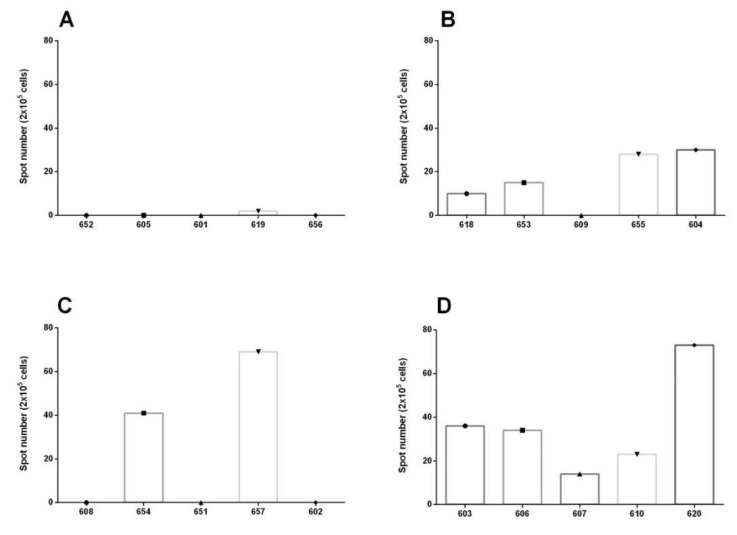
IFNγ reading by ELISPOT. (**A**) Group 1: unvaccinated animals. (**B**) Group 2: animals immunized once five days before challenge. (**C**) Group 3: animals immunized once three days before challenge. (**D**) Group 4: animals immunized once one day before challenge. No statistical differences were detected between the vaccinated groups (Kruskal–Wallis test *p* > 0.05).

Virus-specific T cell IFNγ responses in PBMC were present at the time of challenge in the three vaccinated animals group, but not in the group of unvaccinated animals. The numbers of IFNγ secreting cells were low in all oof the groups. The highest responses were detected in animals in group 3 and group 4 (Figure 4), although no statistical differences were detected between the vaccinated groups (Kruskal–Wallis test *p* > 0.05). The results could demonstrate that vaccination induces an IFN γ response as early as only one day before vaccination. Nevertheless, these higher responses do not correlate with protection, as not all animals with IFNγ responses were protected, nor was an IFNγ response detected in all pf the protected animals that were challenged 5 dpv. This opens an interesting immunological response to E2CD154 that needs to be studied further.

## 4. Discussion

Vaccination against CSF is used to reduce the virus spread and assist in the control of outbreaks or epizooties. Therefore, three of the most important issues in the evaluation of the effectiveness of a CSFV vaccine formulation are whether it is able to prevent clinical disease and virus transmission, as well as achieving the effects of vaccination within the shortest period of time [18]. Only CSFV live attenuated vaccines have been effective in protecting vaccinated pigs as early as five days post-vaccination [19]. 

This study confirms that the Porvac^®^, a subunit vaccine model, has the capability to develop a robust early immune response by five days post-vaccination, which approaches the rate of onset of the rapid protection that is induced by live attenuated vaccines. The results that were obtained in the control group, in which three of the five animals had a delay in the appearance of the clinical signs, could suggest that these animals were affected by a contact infection rather than by the initial viral confrontation. The response of the animals challenged one or three days after vaccination indicates that 40% of the animals were able to effectively respond to the vaccination and they were either protected against the challenge, or that these vaccinated animals were protected from a later contact infection.

Although two out of five animals challenged one or three days after vaccination were protected, the other three pigs showed a more rapid onset of clinical symptoms than controls. This was an unexpected result, and it must be confirmed in future experiments. At present, we can only speculate that, during the first three days after vaccination with Porvac^®^, some of the animals are able to mount an effective innate immune response that helps to instrument a faster onset of the adaptive immune response. However, in other pigs, these early events are still immature or twisted towards an unfavorable immune response.

Several groups have demonstrated that C-strain vaccines confer sterile immunity against challenge at 5 to 7 dpv, with partial protection induced from 1–3 days post-vaccination, as discussed earlier [9,20,21]. Although the ability of the host innate immune system to interact with CSFV replication has been studied in vitro by several groups [21,22,23,24] the innate response factors mediating protection at very early times post-vaccination remain unknown. Notably, CSFV infection has been reported to induce changes in the expression of IFNs [23,24,25]. CSFV possesses mechanisms that hinder the induction and production of IFNs. Furthermore, a direct correlation between the virulence of a CSFV strain and the amount of IFN that is produced during CSFV infection in swine has also been reported [7,26]. C strain vaccinated pigs challenged at six days post-vaccination had a significant higher number of IFN-γ secreting cells when compared to mock vaccinated animals [7]. The direct effect of IFN-γ on CSFV replication in cell cultures was demonstrated. In addition, the direct role of IFN-α in protection against challenge with a virulent CSFV strain has also been reported [27].

E2 subunit vaccines described and commercialized up to now are effective against virus challenge 10 to 14 days after a single dose [28]. The formulation of Porvac^®^ vaccine provided clinical and robust viral protection against high virulent “Margarita” strain at five days post-vaccination. The findings from this study suggest that increased levels of IFN-γ secreting cells after Porvac^®^ vaccination may play a protective role against “Margarita” strain challenge. However, the obtained results do not demonstrate a direct correlation between the levels of interferon gamma secreting cells and the protection of animals for this vaccine model, as some animals that developed a measurable interferon gamma response in virus stimulated PBMC in vitro were not protected from the viral confrontation. 

Previous studies with CSFV MLV informed the presence of IFN-γ secreting cells at 3 dpv [29]. CD4+ and cytotoxic CD8+ T cells were identified as the cellular source of IFN-γ. NK cells are considered one of the most important IFN-γ secreting cells induced in the initial host immune responses in several infections [30]. However, IFNγ secreting γδ T cells were detected in the lymph nodes of C57BL/6 mice as early as 1 dpv with a live Yellow Fever Virus attenuated vaccine [31], while IFN-γ secreting NK cells were detected later at 3 dpv. We hypothesize that CD154 is indirectly triggering a faster IFN-γ secretion by NK cells or T cells, but further work will be needed in order to confirm these results, and to delve into other mechanisms of the innate and early adaptive immune system after vaccination with Porvac^®^, which seems to be cardinal in the shaping of a protective immune response. 

The establishment of a robust antiviral state seems to be crucial in preventing virus replication and the spread of challenge virus in animals receiving CSF vaccines. We will continue to study the immunological mechanism that is induced by this new formulation, which has the advantages of stability and safety that subunit vaccines have as compared to replicative viruses, by examining cytokines expression in PBMC and tissues that are closely related to the entry, replication, and distribution of the virus in the animal. The CD154 (CD40L) has a central role in the development and regulation of adaptive immune response in mammalian and avian species [32]. This molecule has been defined as the most important costimulatory factor for the activation of antigen presenting cells [33,34,35]. Its receptor (CD40) belongs to the same TNF superfamily, and it is a surface protein of B cells, dendritic cells (DCs), macrophages, Langerhans cells, epithelial cells, endothelial cells, and fibroblasts [33]. The binding of CD154 to CD40 on the surface of B cells stimulates the cell proliferation, adhesion, and differentiation. CD40 participation leads to the clonal expansion of B cells, germinal center formation, isotype change, affinity maturation, and the generation of long-lived plasma cells [36]. The detection of an initial neutralizing antibody response as early as at eight days post-vaccination, three days after challenge, may indicate that the CD154 molecule provides an enhancement of B cell activation. To our knowledge, Porvac^®^ is the first reported subunit CSFV vaccine to induce robust protection against a highly virulent CSF virus as early as five days post-inoculation. This suggests that the inclusion of the CD154 extracellular domain into vaccine formulations may enhance protection by stimulating innate and early adaptive immune mechanisms that we aim to continue to examine in future studies.

## 5. Conclusions

Porvac^®^ is the first reported subunit CSFV vaccine to induce robust protection against a highly virulent CSF virus as early as five days post-inoculation.

## Figures and Tables

**Table 1:** Viral isolation. Group 1: unvaccinated animals. Group 2: animals immunized once five days before challenge. Group 3: animals immunized once three days before challenge. Group 4: animals immunized once one day before challenge. Dpc: days post-challenge. (-): negative samples. (+): positive samples. (N/S): no samples.

		Blood					Spleenen	Tonsil	Ileum	Rectal Swab	
Group	Animals	3 dpc	7 dips	14 dpc	21 dpc	28 dpc	At sacrifice			21 dpc	28 dpc
1	652	-	+	-	N/S	N/S	+	+	+	N/S	N/S
	605	-	-	+	N/S	N/S	+	+	+	N/S	N/S
	601	-	-	+	N/S	N/S	+	+	+	N/S	N/S
	619	-	-	+	N/S	N/S	+	+	+	N/S	N/S
	656	-	-	+	N/S	N/S	N/S	N/S	N/S	N/S	N/S
2	618	-	-	-	-	-	-	-	-	-	-
	653	-	-	-	-	-	-	-	-	-	-
	609	-	-	-	-	-	-	-	-	-	-
	655	+	-	-	-	-	-	-	-	-	-
	604	-	-	-	-	-	-	-	-	-	-
3	608	-	-	-	-	-	-	-	-	-	-
	654	+	+	N/S	N/S	N/S	+	+	-	N/S	N/S
	651	-	-	N/S	N/S	N/S	+	+	-	N/S	N/S
	657	-	-	-	-	-	-	-	-	-	-
	602	-	-	N/S	N/S	N/S	+	+	-	N/S	N/S
4	603	-	-	-	-	-	-	-	-	-	-
	606	+	+	N/S	N/S	N/S	+	+	-	N/S	N/S
	607	-	-	-	-	-	-	-	+	-	-
	610	-	+	+	N/S	N/S	+	+	-	N/S	N/S
	620	-	-	N/S	N/S	N/S	+	+	-	N/S	N/S

## Data Availability

Data presented in this study are available on request from the corresponding author.
